# Effects of Transport Inhibitors on the Cellular Uptake of Carboxylated Polystyrene Nanoparticles in Different Cell Lines

**DOI:** 10.1371/journal.pone.0024438

**Published:** 2011-09-19

**Authors:** Tiago dos Santos, Juan Varela, Iseult Lynch, Anna Salvati, Kenneth A. Dawson

**Affiliations:** Centre for BioNano Interactions, School of Chemistry and Chemical Biology, University College Dublin, Belfield, Dublin, Ireland; George Mason University, United States of America

## Abstract

Nanotechnology is expected to play a vital role in the rapidly developing field of nanomedicine, creating innovative solutions and therapies for currently untreatable diseases, and providing new tools for various biomedical applications, such as drug delivery and gene therapy. In order to optimize the efficacy of nanoparticle (NP) delivery to cells, it is necessary to understand the mechanisms by which NPs are internalized by cells, as this will likely determine their ultimate sub-cellular fate and localisation. Here we have used pharmacological inhibitors of some of the major endocytic pathways to investigate nanoparticle uptake mechanisms in a range of representative human cell lines, including HeLa (cervical cancer), A549 (lung carcinoma) and 1321N1 (brain astrocytoma). Chlorpromazine and genistein were used to inhibit clathrin and caveolin mediated endocytosis, respectively. Cytochalasin A and nocodazole were used to inhibit, respectively, the polymerisation of actin and microtubule cytoskeleton. Uptake experiments were performed systematically across the different cell lines, using carboxylated polystyrene NPs of 40 nm and 200 nm diameters, as model NPs of sizes comparable to typical endocytic cargoes. The results clearly indicated that, in all cases and cell types, NPs entered cells via active energy dependent processes. NP uptake in HeLa and 1321N1 cells was strongly affected by actin depolymerisation, while A549 cells showed a stronger inhibition of NP uptake (in comparison to the other cell types) after microtubule disruption and treatment with genistein. A strong reduction of NP uptake was observed after chlorpromazine treatment only in the case of 1321N1 cells. These outcomes suggested that the same NP might exploit different uptake mechanisms to enter different cell types.

## Introduction

Nanomedicine is the application of nanotechnology in innovative ways to develop new approaches and therapies for treatment of diseases, including drug delivery and gene therapy [1]–[6]. In order to utilise NPs to deliver drugs to a target organ or cellular location more effectively, it is essential, as a first step, to understand the distinct endocytic mechanism(s) used by the specific NPs to enter the target cells. From this information, it may be possible to develop approaches to enable NPs to escape the acidic pathway, which often leads NPs to a final localisation in the lysosomes, which is the cellular waste bin [7]. Thus, therapeutic NPs would be much more effective if they could be designed to reach the nucleus (gene therapy) or other organelles involved in disease progression, and if they could deliver their payload directly to the site of action. So far, several distinct cellular uptake pathways for drugs, protein-lipids clusters, virus and bacteria have been described [8]–[12], and many attempts have been made to classify and characterize them, typically according to the proteins involved at the early stages, or via the size of the entities that they take up into cells [13].

Briefly, some aspects of the different uptake pathways are summarised here.

Phagocytosis is restricted to specialized mammalian cells such as neutrophils, monocytes and macrophages, which function to engulf and digest cellular debris and pathogens, and are essential to keep the immune system in a clean and sterile state. Interaction of a pathogen with specific cell surface receptors induces signalling cascades mediated by Rho-family GTPases, triggering polymerization of actin membrane protrusions at the site of ingestion. After internalisation, actin is shed from the phagosome, and a series of fusion and fission events, involving components of the endocytic pathway, culminates in the formation of the mature phagolysosome [14]. Although this process is mainly present in specialised cells, it is known that non-specialised cells can, in rare situations, retain the ability to activate uptake processes which resemble phagocytosis [15].

Other well characterised uptake pathways include: (i) Clathrin-mediated endocytosis (CME), which is a process involving specific receptors that recognize and internalize cargo into “coated pits”, formed by the assembly of a cytosolic coat protein, clathrin, which constitutes the main assembly unit. These coated pits invaginate and pinch off to form endocytic vesicles (i.e. early endosomes), that later mature into late endosomes and fuse with lysosomes. The literature on these pathways is extensive and the structure of clathrin and the clathrin coated pits is well resolved, as is the role of other key proteins involved [16]–[19]. Typical sizes of clathrin coated pits are in the range 60–200 nm diameter [20], [21]. (ii) Caveolae-mediated endocytosis, which involves clustering of lipid raft components on the plasma membrane into so called caveolae, which are flask-shaped invaginations, formed as a result of the interactions of different proteins, mainly caveolin, with the cellular membrane. Caveolae are extremely abundant at the surface of endothelial cells, and internalisation via this pathway is induced by specific ligands such as cholera toxin and simian virus-40, and this is considered to be the predominant pathway of entry for particles above 200 nm [20], [22], [23]. (iii) Other uptake pathways have been classified as clathrin- and caveolae-independent endocytosis. For these pathways, other types of cholesterol-rich microdomains on the plasma membrane are involved, rather than caveolae. These domains are generally referred to as lipid rafts, small structures of 40–50 nm in diameter, that diffuse on the cell surface [24]–[26]. These small rafts can be captured by, and internalized within, an endocytic vesicle and although the regulatory mechanisms for these pathways are still unknown, these alternative endocytic carrier vesicles are known to be rich in glycosyl phosphatidylinositol (GPI)-anchored protein, and can end in the Golgi complex or in recycling endosomes [27], [28]. Literature is growing on these pathways, where specific classes of lipids have been reported to induce membrane curvature to allow the invagination of the plasma membrane, in the absence of proteins like clathrin and caveolin to facilitate the process [29], [30]. (iv) Finally, macropinocytosis involves the internalisation of large areas of the plasma membrane together with significant amounts of fluid, since uncoated vesicles can be bigger than coated ones, thus allowing endocytosis of larger objects (>150 nm). Actin membrane protrusions caused by extensive actin rearrangement, which are triggered by the stimulation of Rho-family GTPases, are a fundamental part of this process [31], [32].

Fundamental questions in the study of the interactions of NPs with cells include whether, and how, NPs map onto these pre-existing cellular uptake mechanisms. As a result of their nanoscale size, which is similar to that of many of the protein-lipid clusters (lipoprotein complexes) routinely transported into and out of cells, and their ability to interact with lipoproteins [33], [34], NPs are now known to have the ability to enter cells, interacting with the cellular machinery through active, energy-dependent processes [7], [35]–[42].

In the present paper, we seek to investigate by which endocytic mechanism(s) negatively charged carboxylated-modified polystyrene NPs, of two sizes representative of typically endocytotic cargoes (40 nm, 200 nm), are internalized by a panel of different cell types, including HeLa tumoral epithelial cells from Cervix, commonly used to study cell biology of uptake mechanisms [43]–[45], A549 endothelial cells from lung carcinoma, widely applied in toxicity studies for lung exposure scenarios [46], [47], and 1321N1 (glial cells from brain astrocytoma), which are a good model for impact in the central nervous system [48], [49]. The uptake of the model polystyrene NPs was systematically studied in the presence and absence of a series of pharmacological inhibitors of different aspects of endocytosis. Despite showing poor specificity, these tools are useful and widely used in cell biology, and are now being applied to the question of NP-cell interactions[18], [50], [51]. The inhibitors used in this work were: genistein, an inhibitor of tyrosine kinases involved in caveolae-mediated endocytosis [52], [53]; chlorpromazine, which inhibits clathrin disassembly and receptor recycling to the plasma membrane during clathrin-mediated endocytosis [54]; nocodazole, a microtubule-disrupting agent [55], [56]; and cytochalasin A, an actin-disrupting agent that is used widely to study the role of actin filaments in different biological systems. The efficacy of the inhibition of NP uptake in the different cell lines was quantified by flow cytometry relative to the control cells in the absence of the pharmacological inhibitors.

## Results and Discussion

Carboxylated-modified polystyrene (PS-COOH) NPs with nominal sizes of 40 nm and 200 nm were chosen as model particles in order to understand the mechanism of internalisation in different cell lines, due to their wide availability to the scientific community, and the fact that they are fluorescently labeled in a manner that we have previously shown is suitable for uptake studies (i.e. they do not elute significant quantities of free dye under cell culture conditions over the timescale of uptake studies) [42]. We focused the work on these NP sizes, as being representative of typical naturally occurring “nanoparticles” such as low-density lipoprotein (LDL), very-low-density lipoprotein (VLDL) etc (which have a diameter around 30–50 nm) and larger objects which are, however, still below the limit of specialized phagocytosis [8], [10], [13], [57]. Electron Microscopy studies on the same NPs confirmed that the manufacturer's stated particle sizes corresponded mainly to their nominal size [58]. As reported in Table 1, the particles were monodisperse in size when measured in PBS buffer, and no strong aggregation was found when particles were dispersed in complete MEM medium (cMEM supplemented with 10% foetal bovine serum, FBS). Zeta potential measurements in Dulbecco's Phosphate Buffered Saline (DPBS) and cMEM, showed that the particles have a negative surface charge, with a zeta potential of −26 mV and −28 mV for the 40 nm and 200 nm PS-COOH NPs respectively in PBS, which decreased to −10 mV and −3 mV respectively in cMEM, due to serum protein adsorption onto the NPs. The lower zeta potential in cMEM did not result in NP instability and aggregation, probably due to steric hindrance from the proteins adsorbed on the NP surface in cMEM. All cellular studies were performed using the full panel of cell lines. Flow cytometry has been used to measure average cell fluorescence intensity after uptake of 20 µg/mL fluorescently labeled NPs. A typical profile of uptake as a function of exposure time is shown in Figure S1 for the two NPs used in this study in HeLa cells, together with representative confocal images of cells treated in the same way, which confirmed that the NPs accumulate inside the cells. Similar profiles have been obtained for all the investigated cell lines (data not shown) and indicate that cell fluorescence increases with time, as NPs accumulate in the cells (having confirmed the absence of free dye that elutes from the particles) [42]. We then compared the cell fluorescence levels at a given NP exposure time (2 h) in the absence or presence of the different transport inhibitors (see Materials and Methods for details). Thus, we report the results as the percentage of NP uptake in cells treated with the different inhibitors, with respect to NP uptake in untreated (normal) control cells. This also implies that the results are not affected by the different fluorescence intensities of the two different NP sizes. It is important to stress that the exposure protocols (as detailed in the Methods section) have been designed to ensure control of each step: Figure S2 shows, as an example, the optimization performed to ensure particle removal from the external side of the cell membrane, prior to cell fluorescence assessment by flow cytometry.

**Table 1:** PS-COOH NP characterization in PBS buffer and complete medium (cMEM, containing 10% foetal bovine serum).

Carboxylate modified polystyrene NPs	Mean hydrodynamic diameter (nm)^a^		PDI^b^		Zeta Potential (mV)		
	PBS	cMEM	PBS	cMEM	PBS	cMEM	
PS 40 nm	48±1	59±1	0.015	0.123	-26±3	-10±2	
PS 200 nm	247±3	269±1	0.035	0.061	-28±2		-3±7

a z-average hydrodynamic diameter extracted by cumulant analysis of the data (number distributions).

b Polydispersity index from cumulant fitting.

Polydispersity index.

### Assessment of energy dependence and transport inhibition of the uptake of PS-COOH NPs

**Figure 1:**
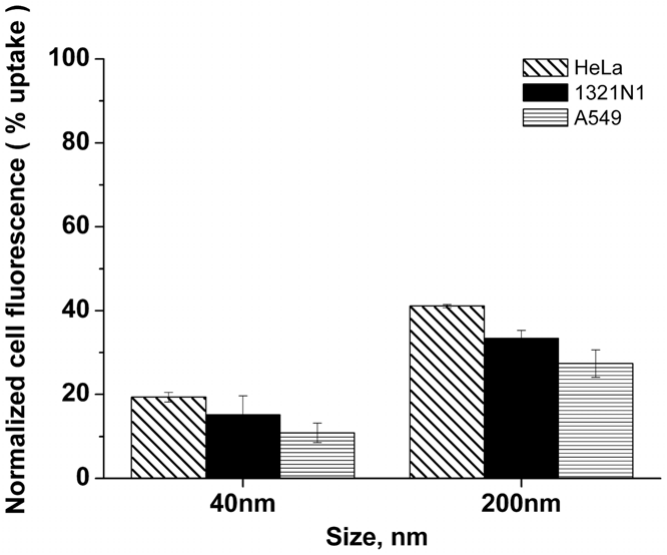
Energy dependent internalisation of 40 nm and 200 nm PS-COOH NPs into various cell lines. Cells were pre-incubated for 30 min at 4°C, and subsequently exposed to the NPs, also at 4°C for 2 h, followed by fixation and FACS analysis. Mean values and standard deviations of three independent duplicate experiments are given.

In order to determine whether NP uptake was an active or passive process, cell cultures were incubated in parallel at 37°C (normal cell culture conditions) and at 4°C. It is known that several proteins and enzymes are sensitive to temperature, thus active processes are inhibited by lowered temperatures [59], [60]. Exposure to NPs at 4°C resulted, as expected, in a very strong inhibition of endocytosis of both particle sizes in all three cell types (Figure 1): in HeLa cells, the average cellular fluorescence was reduced by about 80% for the 40 nm particles and 60% for the 200 nm NPs, in respect to the uptake at 37°C; in A549 cells the uptake was reduced by about 90% and 70% respectively for the 40 nm and 200 nm particles, and for 1321N1 cells 82% and 62% respectively. Similar experiments with SiO_2_ NPs of several sizes showed the same behavior, with NP uptake being highly energy dependent [7]. Although lowering the culture temperature will affect the diffusion of the NPs, we have demonstrated previously that diffusion is not the rate limiting step for NP uptake in cells [42], and thus we can confirm that the reduced uptake is a consequence of inhibition of active transport, rather than a consequence of reduced NP diffusion (the same applies for the different diffusion rates of NPs of different sizes). Moreover, a trend in the inhibition for the different particle sizes can be recognized for all cell lines, with the uptake of the smaller particles (40 nm PS-COOH) showing a stronger reduction in energy depleted conditions, compared with that of the 200 nm PS-COOH NPs.

**Figure 2:**
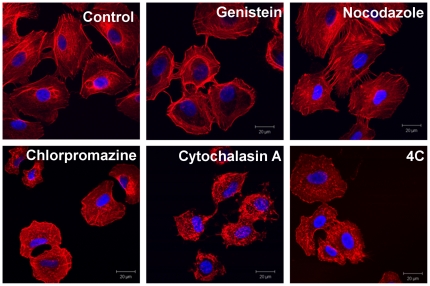
Confocal images of A549 cells showing the F-actin morphology in normal control cells and, following incubation with each of the different inhibitors at 37°C or 4°C for 2 h30 min. (Blue DAPI stained nuclei, Texas red-phalloidin stained actin filaments, Magnification 63X). The scale bar corresponds to 20 µm.

One important requirement when using endocytosis inhibitors (genistein, chlorpromazine, and nocodazole) is that they should not affect the actin cytoskeleton, since reorganization of the actin filaments may, as a side effect, impact on uptake processes which do not directly involve actin and may alter the function of various plasma membrane proteins [61]–[63], thereby confounding the data and leading to multiple effects occurring simultaneously. Figure 2 shows the organization of the actin filaments in the A549 cell line after 2 h of treatment with each of the four inhibitors used in the study (no NPs present). Even though these inhibitors always affect the cell population viability (as discussed further below), we can observe that the actin filaments maintain their overall structure when exposed to each of the inhibitors used, with the exception of cytochalasin A, a known actin-disrupting agent, which was used with the specific purpose of investigating the role of actin filaments in NP uptake. A similar trend was observed in the other cell lines (Figure S3a and S3b). All of the inhibitors were used according to the manufacturer's protocols, which are also frequently reported in the literature for different cell lines in order to achieve transport inhibition [64]–[66]. When possible, transport of molecules known to selectively enter cells via a specific pathway were used as positive controls to confirm that the required inhibition was achieved, such as transferrin and cholera toxin B for clathrin and caveolae mediated endocytosis, respectively (as shown in Figure S4). However, this was not possible in all cases due to the lack of specificity of these molecules.

**Figure 3.Cell:**
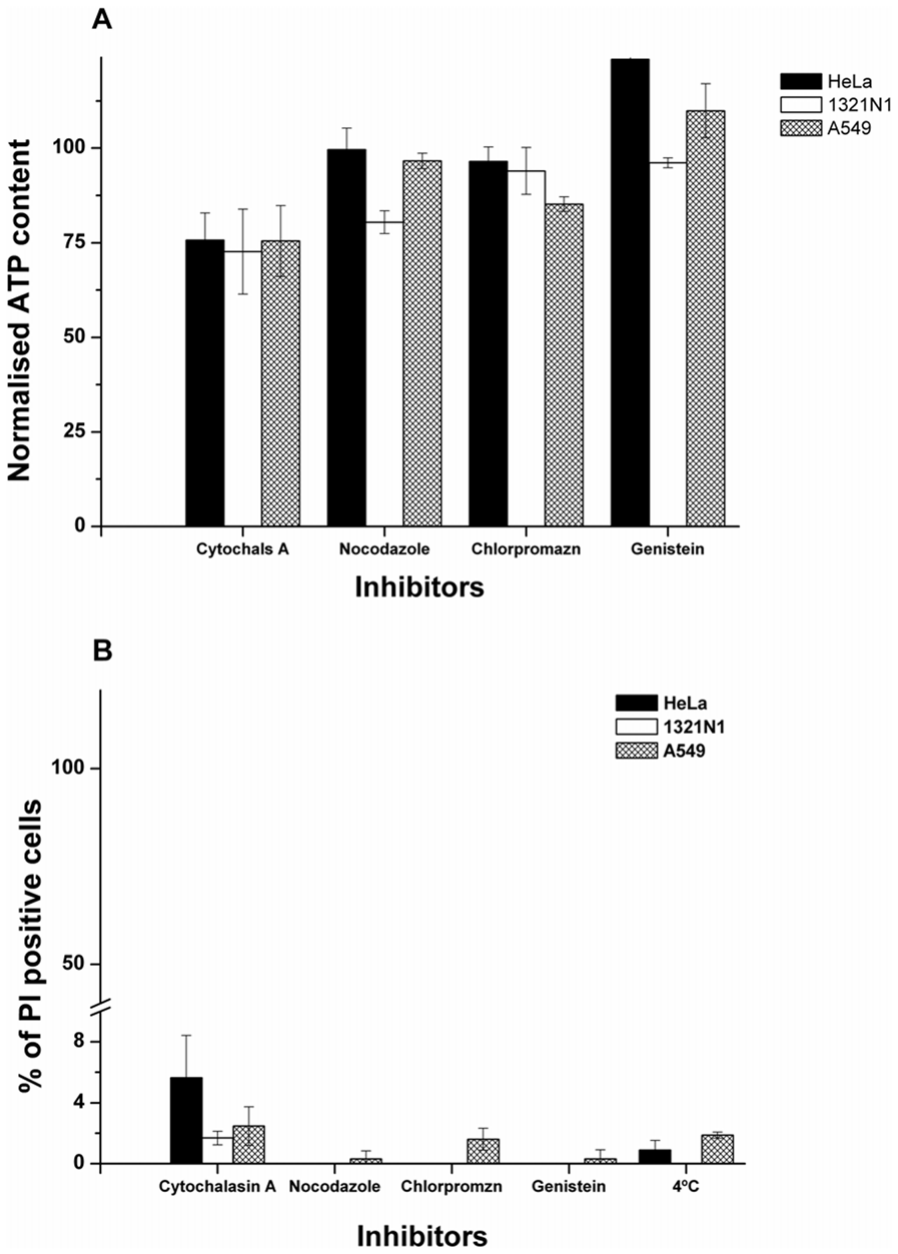
Cell viability as determined by a) ATP assay to measure ATP content in cells and b) propidium iodide staining as a measure of membrane damage, following exposure to each one of the different inhibitors for 2 h30 min. Results are presented as a percentage of ATP levels and PI positive cells, with respect to control (untreated) cells, averaged between 2 independent replicas of 3 independent experiments.

NP uptake was measured only at short exposure times, since it has been previously reported that blocking one uptake pathway can result in activation of other endocytic mechanisms, which again confounds the data [67]. Thus, a balance must be found between allowing sufficient time for detectible levels of NPs to enter the cells, and for the transport inhibitors to reduce endocytosis, without inducing too-severe side effects in terms of toxicity from these molecules. On this basis, it is also essential to evaluate the cellular toxicity of each of the pharmacological inhibitors, as a quality control step prior to their co-incubation with the NPs. Thus, the cell viability of each of the cell lines was assessed after 2 h of exposure to each of the different inhibitors, by measuring ATP levels and by propidium iodide (PI) staining. PI only penetrates cells with increased cell membrane permeability, thus accumulates in apoptotic or necrotic cells, and thereby the percentage of PI positive cells, which can be assessed by flow cytometry, can be used as a measurement of cell damage. Results are shown in Figure 3 and indicated that no strong cell damage and cell loss after treatment with the four different pharmacological inhibitors was found for all cell lines used in this study.

**Figure 4:**
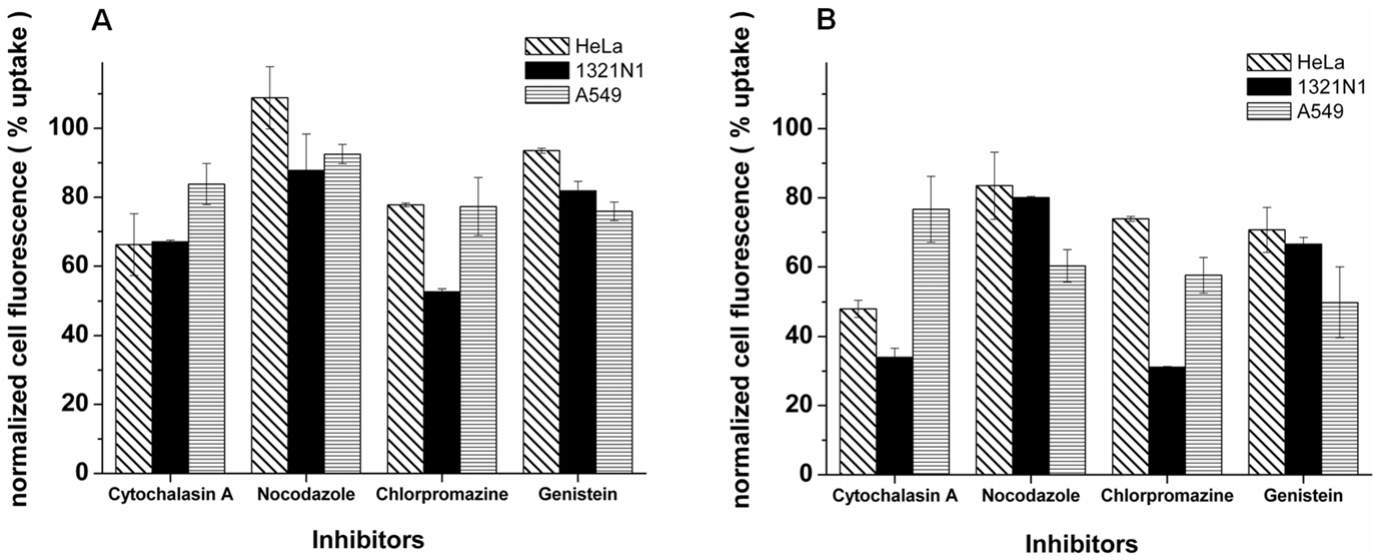
Effect of transport inhibitors on the internalisation of a) 40 nm and b) 200 nm PS-COOH NPs into the various cell lines. Cells were pretreated with each one of the inhibitors (cytochalasin A, nocodazole, chlorpromazine and genistein) for 30 min, followed by 2 h of exposure to the NPs, in the presence of the same inhibitor, then fixed and analyzed by FACS. Mean values and standard deviations of three independent duplicate experiments are given. Results are reported as the percentage of uptake relative to untreated cells exposed to NPs. This was calculated by dividing the cell fluorescence after NP uptake in drug treated cells by that of control cells which were not treated with the inhibitors.

The NP uptake results after treatment with the transport inhibitors are shown in Figure 4 for the different cell lines investigated (with the results for the 40 and 200 nm PS-CPPH NPs respectively in panel a and b).

### The effect of microtubules and F-actin on the uptake of PS-COOH NPs

The cytoskeleton plays an important role in multiple cellular events, including endocytosis and trafficking of endocytosis vesicles. For this reason, we have investigated the role of two of the major constituents of the cytoskeleton structure, microtubules and F-actin, in NP uptake and internalisation. Although both microtubules and F-actin are essential in most of the uptake mechanisms described above, it is important to evaluate how strongly NP uptake depends on the presence of these structures. As mentioned above, cytochalasin A can disrupt F-actin polymerization, and as such, has been used to investigate the role of F-actin in cellular uptake of PS-COOH NPs. As shown in Figure 4a, in the case of HeLa and 1321N1 cells, cytochalasin A inhibited the endocytosis of 40 nm PS-COOH particles by about 30% relative to the control cells. For the 200 nm PS-COOH NPs, the effect of cytochalasin A was stronger, with a 50% reduction of uptake in HeLa cells and a 65% reduction of uptake in 1321N1 cells, relative to the controls (Figure 4b). This suggests a strong involvement of the actin filaments in the uptake process of PS-COOH NPs in these cell lines and especially for the larger PS-COOH particles. Interestingly, results for A549 cells showed a smaller inhibition of uptake, with only ∼20% reduction for both particle sizes, indicating a smaller dependency of PS-COOH NP uptake on F-actin for this particular cell line. This result could also be explained by a different efficiency of the inhibition induced by cytochalasin A in the different cell lines, however the images shown previously (Figure 2) seem to exclude this possibility, as strong actin depolymerisation was evident for all the cells used in the study, including the A549 cells (Figures 2, S3a and S3b)).

It has been reported that microtubules are also involved in the endocytic process, and thus nocodazole was used as a microtubule disruptor in order to assess the influence of microtubules on NP internalisation by the panel of cells. The effects of this treatment were not as strong as with actin depolymerisation. Nocodazole was found to inhibit endocytosis of larger particles (200 nm PS-COOH) primarily, with the highest inhibition observed in the A549 cell line, where uptake was reduced by ∼40% relative to control cells (Figure 4b). In the case of HeLa and 1321N1 cells, the inhibition of uptake of 200 nm PS-COOH NPs was slightly less, with uptake being reduced by ∼20% relative to normal controls (Figure 4b). For the smaller NPs (40 nm PS-COOH), the inhibition was only 5–10% relative to the normal control cells in the case of A549 and 1321N1 cells, and no reduction of uptake was observed in HeLa cells (Figure 4a). These results suggest that microtubules are mostly involved in the uptake of larger particles, and are particularly relevant in the uptake of PS-COOH particles by A549 cells. These results are in agreement with Dausend *et al*. (2008) who found that uptake of 120 nm negatively charged polystyrene NPs by HeLa cells was not inhibited by nocodazole.

### Mechanism of PS-COOH NP internalisation: clathrin vs caveolin

It is well established that transferrin (Tf) is a ligand exclusively internalized via the clathrin-coated-pit endocytosis pathway, and thus its uptake is expected to be affected by treatment with chlorpromazine, an inhibitor of clathrin-mediated endocytosis. For this reason, we used transferrin as a positive control to confirm the inhibition of this particular endocytotic pathway upon treatment with chlorpromazine [54]. The results are shown in Figure S4 and, as expected, indicate a very strong reduction of Tf uptake in chlorpromazine treated cells, thus confirming the applied inhibition protocol. Treatment with chlorpromazine resulted in a strong inhibition of PS-COOH NP uptake in the 1321N1 cell line, with the larger particles having a strong (70%) decrease in uptake, relative to normal control cells, compared with a 50% inhibition of uptake for the 40 nm PS-COOH particles (Figure 4). Although this trend in the level of inhibition for the two PS-COOH particle sizes was found also in A549 cell line, it is noteworthy that in HeLa cells the levels of inhibition were quite similar with both particles. Rejman *et al*. (2004), using similar negatively charged polystyrene NPs, with sizes between 50 and 200 nm in B16 cells (mouse melanoma cell line), showed instead that chlorpromazine treatment affected more strongly the uptake of the smaller NPs, which is the opposite to what we found in the cell lines used in this study. Moreover, Dausend *et al*. (2008) showed no significant inhibitory effect of chlorpromazine on the uptake of 120 nm negatively charged polystyrene NPs by HeLa cells.

As described above, receptor-associated tyrosine-specific protein kinases are reported to be involved in lipid raft-dependent endocytosis, thus genistein, which is a specific inhibitor of these proteins, can be used to study the involvement of lipid raft-dependent uptake mechanisms in the uptake of PS-COOH NPs by our panel of cells. It is noteworthy that the concentration of genistein applied in the present study was not sufficient to significantly inhibit the uptake cholera toxin B (also shown in Figure S4). This confirms a major problem for this kind of pharmacological drug, which is the lack of specific positive controls for each of the distinct endocytic pathways [68]. However NP internalisation was strongly reduced by genistein in A549 cells (Figures 4a and 4b), especially for the 200 nm PS-COOH NPs, with a decrease in the uptake of 50% relative to normal control cells. This suggests that there is a strong participation of lipid raft-associated receptors in the endocytosis of the 200 nm PS-COOH particles for this cell line. In addition, we can again observe a trend for the different cell lines, with a higher inhibition for the bigger (200 nm) particles, when compared to the 40 nm PS-COOH NPs. These results are in agreement with Rejman *et al*., who suggested that caveolae flask-shaped invaginations are able to internalize larger particles more easily than clathrin coated pits (with a structure that is restricted by the triskeletal structure of clathrin). Although lacking a clear mechanistic explanation as yet, electrostatic interactions and kinetics of internalisation could explain why, despite the size of the caveolae being normally 60–80 nm, genistein has such a big effect in the uptake of 200 nm NPs.

A summary of all the results obtained with the different transport inhibitors in the different cell lines is given in Table 2, together with some of the results available in the literature for similar systems. The levels of inhibition of uptake (NPs and positive control molecules) observed with the different drugs are presented there as a percentage with respect to the uptake obtained in normal cells exposed to NPs (or to positive control molecules) under the same conditions (so that the level of inhibition in normal control cells is 0%).

**Table 2:** Levels of inhibition of the uptake of 40 nm and 200 nm PS-COOH nanoparticles (and positive controls) in cells exposed to the different inhibitors in respect to the uptake in untreated cells.

Level of inhibition (%) relative to control						
		Cytochalasin A	Nocodazole	Chorpromazine	Genistein	4°C
**HeLa**	40 nm NPs	34±9	0±9	23±1	7±1	80±1
	200 nm NPs	52±3 (50)[45]	17±10 (0)[45]	26±1 (0)[45]	30±7	60±1 (60)[45]
	Transferrin	-	-	94±1 (80)[45]	-	-
	Cholera toxin B	-	-	-	-	-
**A549**	40 nm NPs	17±6	8±3	23±8	24±3	90±2 (70)[7]
	200 nm NPs	23±10	40±5	43±5	50±10	70± 3 *(70)* [*7*]
	Transferrin	-	-	90	-	-
	Cholera toxin B	-	-	-	14±16	-
**1321N1**	40 nm NPs	33±1	13±10	47±1	19±3	82±5
	200 nm NPs	66±3	20	69	33±2	62±2
	Transferrin	-	-	96	-	-
	Cholera toxin B	-	-	-	-	-

Results are presented as a percentage in respect to the uptake in normal (untreated) cells under the same conditions and averaged between 2 independent replicas of 3 independent experiments. In *italic* and brackets some comparative data from experiments performed by other researchers, in similar systems, with respective references.

Different outcomes were observed across the different cell lines and in several cases the treatment with the inhibitors showed only a small effect on nanoparticle uptake. In the case of HeLa cells, the strongest effects were observed after depolymerisation of the actin filaments by treatment with cytochalasin A, suggesting a central role for actin in NP uptake in this cell line. Actin is required for several endocytic pathways, including clathrin mediated endocytosis, however the chlorpromazine treatment, which also affects this pathway, did not inhibit NP uptake in HeLa cells to a similar extent. In 1321N1 cells, NP uptake was also strongly affected by actin depolymerisation. These cells showed a significant inhibition of NP internalisation after treatment with chlorpromazine for both investigated particle sizes, with stronger effects being observed for the larger particle size, suggesting that both actin and clathrin might be implied in the NP uptake mechanisms. Finally, the A549 cells were the least affected by actin depolymerisation, while only for this cell type NP uptake was affected by microtubule depolymerisation and treatment with genistein to a small extent. This suggests a bigger role of microtubules and lipid rafts for NP internalisation in A549 cells, with the stronger effects observed, also in this case, for the larger particle size.

In conclusion, NPs have the capacity to interact with the cellular machinery and can enter very different cell types with ease. The data presented here highlight the complexity and interplay between the mechanisms by which different cell lines are able to endocytose NPs, and suggest that the same NP might be internalised via different pathways in different cell types. NP uptake was most effectively reduced when active processes were inhibited, and this indicated that cells need to spend energy in order to internalize NPs. None of the pharmacological treatments could fully inhibit NP uptake and this leaves open the possibility that within one cell type, they use multiple pathways simultaneously to internalise the same NP. The results clearly indicate the importance of careful analysis and individual interpretation for each cell-NP system, and the need to build up a database of information regarding the responses of different cell types to common NPs, in order to begin to construct patterns and trends in cellular responses to NPs. It is clear that great care and significant additional work must be undertaken in order to be able to fully investigate NP uptake mechanisms and to safely use NPs for nanomedicine applications.

## Materials and Methods

### Cell culture

Human glial astrocytoma 1321N1 cells (passage 2–10 after defrosting from liquid nitrogen)were cultured at 37°C in 5% CO_2_ in Dulbecco's Modified Eagle's Medium (DMEM) supplemented with 10% foetal bovine serum (FBS, Gibco) and 1% penicillin/streptomycin (Invitrogen Corp.).

Human lung epithelium A549 cells (passage 1–30 after defrosting from liquid nitrogen; original batches at passage number 105 or 82) were cultured at 37°C in 5% CO_2_ in Minimum Essential Medium (MEM, with additional L-Glutamine) supplemented with 10% foetal bovine serum (FBS, Gibco), 1% penicillin/streptomycin (Invitrogen Corp.), and 1% MEM non-essential amino acids (HyClone).

Human cervix epithelium HeLa cells (passage 5–10) were cultured at 37°C in 5% CO_2_ in Minimum Essential Medium (MEM, with additional L-Glutamine) supplemented with 10% foetal bovine serum (FBS, Gibco) and 1% penicillin/streptomycin (Invitrogen Corp.).

All cell lines were confirmed to be mycoplasma negative using the mycoAlert kit (Lonza Inc. Allendale, NJ) and were tested monthly.

### Nanoparticles

Red dye-loaded (Excitation/Emission wavelengths: 580–605) carboxylate-modified polystyrene NPs (Fluospheres® size kit, Invitrogen) were used without further modification or purification. Sizes used in this work were 40 nm, 200 nm. All stock solutions were stored at 4°C, and used within 1 year of purchase, in order to ensure their stability.

NP dispersions were prepared by diluting the concentrated stock solutions into the complete medium (cMEM) used for cell culture at room temperature, with or without inhibitor drugs, immediately prior to the experiments on cells, with an identical time delay between diluting and introducing to the cells for all experiments. The medium was kept at room temperature and not pre-warmed to 37°C to ensure better NP dispersions. Before sampling, NPs were vigorously mixed by vortexing, as recommended by the company.

The mean size and surface charge of NPs were determined using a photon correlation spectrophotometer (Malvern Zetasizer Nano ZS). Measurements were performed at 25°C in DPBS and in MEM. The particle size distribution and zeta potential data are presented in Table 1.

### Flow cytometry analysis of NPs uptake: energy dependence and inhibition

1.25×10^5^ cells were seeded in individual 30 mm tissue culture dishes (Greiner Bio-one), and incubated for 24 h prior to addition of particles. Each step from NP exposure to cell to sample preparation for flow cytometry has been optimized in order to ensure reproducibility of the data and quantitative information to be obtained. Several artifacts could arise from this kind of experiments without such optimizations and control. Thus, after 24 hrs, cells were pre-incubated for 30 min with the different drugs at the following concentrations: chlorpromazine hydrochloride 10 µg/ml, cytochalasin A 5 µg/ml, genistein 200 µM and nocodazole 20 µM (all from Sigma). Energy dependence experiments were performed by pre-incubating the cells at 4°C for 30 min prior to exposure to NPs. After this pre-incubation, NPs (final concentration 20 µg/ml) were added and incubated for 120 min, either in the presence of the drugs or at 4°C. Negative controls, i.e., cells without the presence of drugs and/or NPs were also carried out. After this incubation time, the medium was removed and the samples were washed with DPBS, in order to ensure particle removal from the outer cell membrane. As an example of the protocol optimization, figure S2 shows the fluorescence intensity of several PBS washes of the cell plate, following particle removal. The results indicated that a significant residual amount of NP could be detected in the first washes after particle removal. Thus we set the number of washes with PBS to a minimum of 3, to optimally remove NPs adhering on the cell membrane or on the plate, which could affect following steps and the quantification of cell fluorescence intensity by flow cytometry. Cell were then harvested with 0.05% trypsin/EDTA 1x, and cell pellets were fixed with 4% formalin solution neutral buffered (Sigma) for 20 min and re-suspended with constant volumes of DPBS before cell-associated fluorescence (10,000–40,000 cells per sample) was detected using a CyAn ADP (DAKO) flow cytometer. In all the cases, to ensure reproducibility, the waiting time between sample preparation and measurement was kept constant, at a minimum of 1.5 h, to ensure complete stability of the sample. Strong modifications of the side scattering, forward scattering and fluorescence intensities were detected in the 1st h, after which all these parameters were more constant with time, but still changing slightly. Samples were stored in darkness at 4°C before measurements. The results are reported as the mean of the distribution of cell fluorescence intensity obtained by measuring 15000 cells, averaged between 2 independent replicas of 3 independent experiments. Error bars are the standard deviation between these independent experiments. Flow cytometry was carried out in the Flow Cytometry Core Facility at the Conway Institute, University College Dublin.

### Cell viability in the presence of the inhibitors: ATP and propidium iodide assays

Cells were seeded in white, clear bottomed 96 well micro-assay-plates, (Greiner bio-one) at 10^4^ cells/100 µl/well (n = 6) and incubated for 24 h. After 24 hrs, cells were incubated for 120 min with 50 µl of medium containing one of the inhibitors at the concentrations mentioned above. Normal cells were used as the negative control. ATP content was measured in accordance to the protocol of the CellTiter-GloTM luminescent cell viability assay kit (Promega). Briefly, 50 µl of assay reagent was added to the wells and mixed for 2 min at room temperature. After 10 min, intracellular ATP content was measured using a Varioskan Flash plate reader (ThermoFisher Scientific). Normalisation of ATP content was calculated using the following equation: Normalised ATP content (in percentage)  =  [value (cells exposed to drug)/value (cells untreated with drugs)]×100. For the propidium iodide assay, 1.25×10^5^ cells were seeded in individual 30 mm tissue culture dishes (Greiner Bio-one), and incubated for 24 h prior to addition of drugs. After 24 hrs, cells were incubated for 120 min in the presence of one of the drugs at concentration described before. After this incubation time, the medium was removed and the samples were washed thrice with DPBS and harvested with 0.05% trypsin/EDTA 1x. Cell pellets were re-suspended with 0.5 ml of DPBS and kept ice-cold until measurements were performed. 5 µl of propidium iodide was added per sample, and this was vortexed for 1 min and cell-associated fluorescence (10,000-30,000 cells per sample) was detected using a CyAn ADP (DAKO) flow cytometer. The % of PI positive cells was calculated using the following equation  =  [% of PI positive cells (cells exposed to drugs) - % of PI positive cells (cells untreated with drugs)].

### Fluorescence microscopy

For confocal microscopy, 3.0×10^4^ cells were seeded onto glass slides (Falcon, 4 well slides) and incubated for 24 h prior to addition to particles. The cell number was set to ensure a cell density comparable to the flow cytometry experiments and, in order to keep all parameters affecting the experiment constant, the same protocols were used for exposure to particles, sample preparation and cell fixation. Thus, particle dispersions were prepared in cMEM at room temperature just before addition to the cells and, after particle exposure, medium was removed and all samples were washed thrice with DPBS, fixed for 20 min with 4% formalin solution neutral buffered, and the nucleus stained with 4′,6-diamidino-2-phenylindole (DAPI blue). For F-Actin staining, after exposure to one of the inhibitors, and fixation as described above, cells were permeabilised with 0.1% Saponin (Sigma Aldrich) for 5 min before staining the actin filaments by incubation with Texas-red phalloidin (Invitrogen Corp.) for 20 min.

A confocal microscope (Carl Zeiss LSM 510 UVMETA, Thornwood. NY) was used to capture images of the intracellular environment and the sub-cellular localisation of the fluorescent polymeric NPs. For multi-color microscopy, samples were excited with 364 nm (blue channel), 488 nm (green channel), and 543 nm (red channel) laser lines, and images were captured by multi-tracking to avoid bleed-through between the fluorophores. To achieve the necessary signal to noise ratio, while obtaining the thinnest possible optical slices, the pinhole diameters were set to less than 1 airy unit. After adjustment of the pinholes of both lasers to obtain the same optical slices, the optimal optical section that fulfilled our criteria was in the range 0.7–0.8 µm at 63X. The gain and offset for the different channels were kept constant along the full series of experiments in order to allow quantitative comparison of the cell fluorescence intensities.

## Supporting Information

Figure S1 **Kinetics of uptake of 40 nm and 200 nm PS-COOH NPs by HeLa cell line, as determined using flow cytometry over 24 hrs.** NP concentration is 20 µg/ml. Averaged mean values of triplicate experiments are given. Inserts: confocal images, showing 40 nm and 200 nm PS-COOH NPs internalized by HeLa cells (Blue DAPI stained nuclei, FITC-phalloidin stained actin filaments, and NPs in red. Magnification 63X). The scale bar corresponds to 20 µm.
(TIF)

Figure S2 **Fluorescence emission of the PBS washes of HeLa cells following exposure to NPs.** PBS washes were used to remove non internalised nanoparticles adhering on the cell membrane prior to assessment of the cell fluorescence intensity by flow cytometry. Similar data were obtained for the other cell lines. From this data it is clear that 3 washes with PBS are sufficient to ensure that the remaining cell fluorescence is from nanoparticles that have been internalised by the cells.
(TIF)

Figure S3 **Confocal images of a) 1321N1 and b) HeLa cells, showing their F-actin morphology, after incubation with the different inhibitors at 4°C for 2 h30 min (Blue DAPI stained nuclei, Texas red-phalloidin stained actin filaments.** Magnification 63X). The scale bar corresponds to 20 µm.
(TIF)

Figure S4 a) Effect of chlorpromazine on internalization of transferrin into different cells. Cells were pre-treated with chlorpromazine for 30 min, followed by 10 min of exposure to Alexa fluor® 488 labelled transferrin in the presence of chlorpromazine, before being fixed and analyzed by FACS. b) Effect of genistein on internalization of cholera toxin B into A549 cells. Cells were pre-treated with genistein for 30 min, followed by 20 min of exposure to Alexa fluor® 488 labelled cholera toxin B in the presence of genistein, before being fixed and analyzed by FACS. Mean values and standard deviations of duplicate samples are given. Results are reported as % uptake relative to the control cells which were not treated with the inhibitor.
(TIF)
