# Supplementary figures and images for: Effects of Transport Inhibitors on the Cellular Uptake of Carboxylated Polystyrene Nanoparticles in Different Cell Lines
Source: PLoS One. 2011 Sep 19;6(9):e24438. doi: 10.1371/journal.pone.0024438 (PMC3176276; doi:10.1371/journal.pone.0024438)

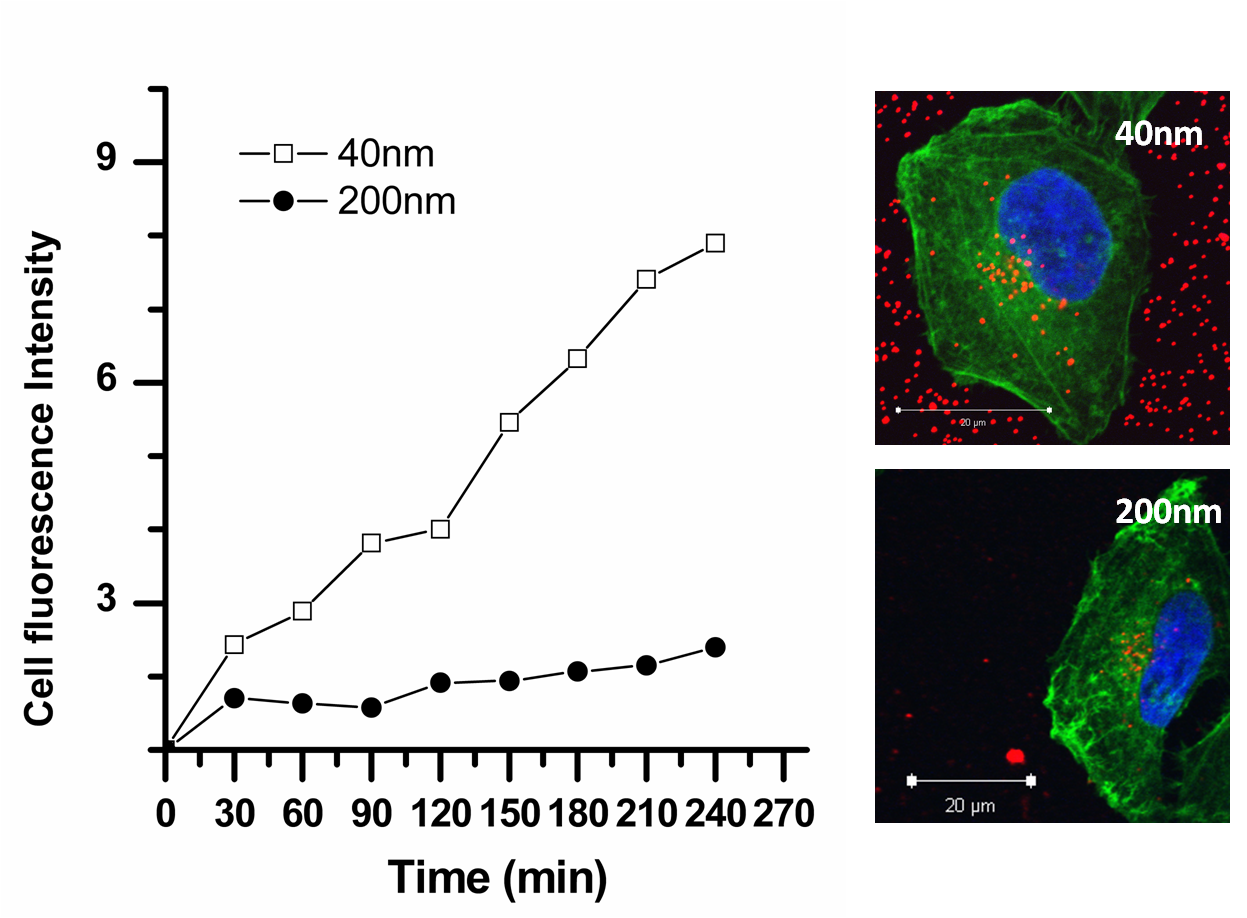

Supplement: Figure S1 — Kinetics of uptake of 40 nm and 200 nm PS-COOH NPs by HeLa cell line, as determined using flow cytometry over 24 hrs. NP concentration is 20 µg/ml. Averaged mean values of triplicate experiments are given. Inserts: confocal images, showing 40 nm and 200 nm PS-COOH NPs internalized by HeLa cells (Blue DAPI stained nuclei, FITC-phalloidin stained actin filaments, and NPs in red. Magnification 63X). The scale bar corresponds to 20 µm. (TIF) [file pone.0024438.s001.tif]

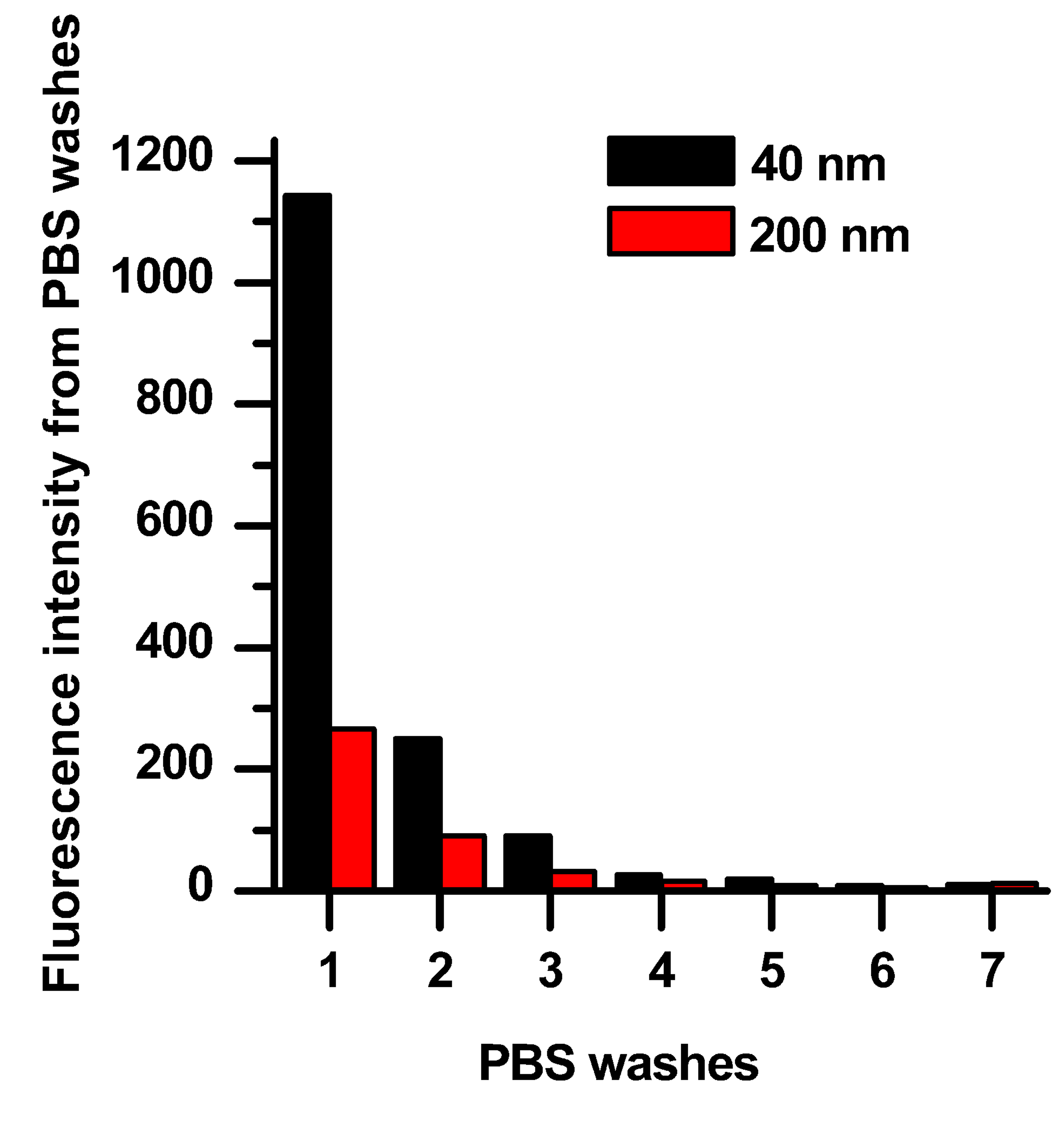

Supplement: Figure S2 — Fluorescence emission of the PBS washes of HeLa cells following exposure to NPs. PBS washes were used to remove non internalised nanoparticles adhering on the cell membrane prior to assessment of the cell fluorescence intensity by flow cytometry. Similar data were obtained for the other cell lines. From this data it is clear that 3 washes with PBS are sufficient to ensure that the remaining cell fluorescence is from nanoparticles that have been internalised by the cells. (TIF) [file pone.0024438.s002.tif]

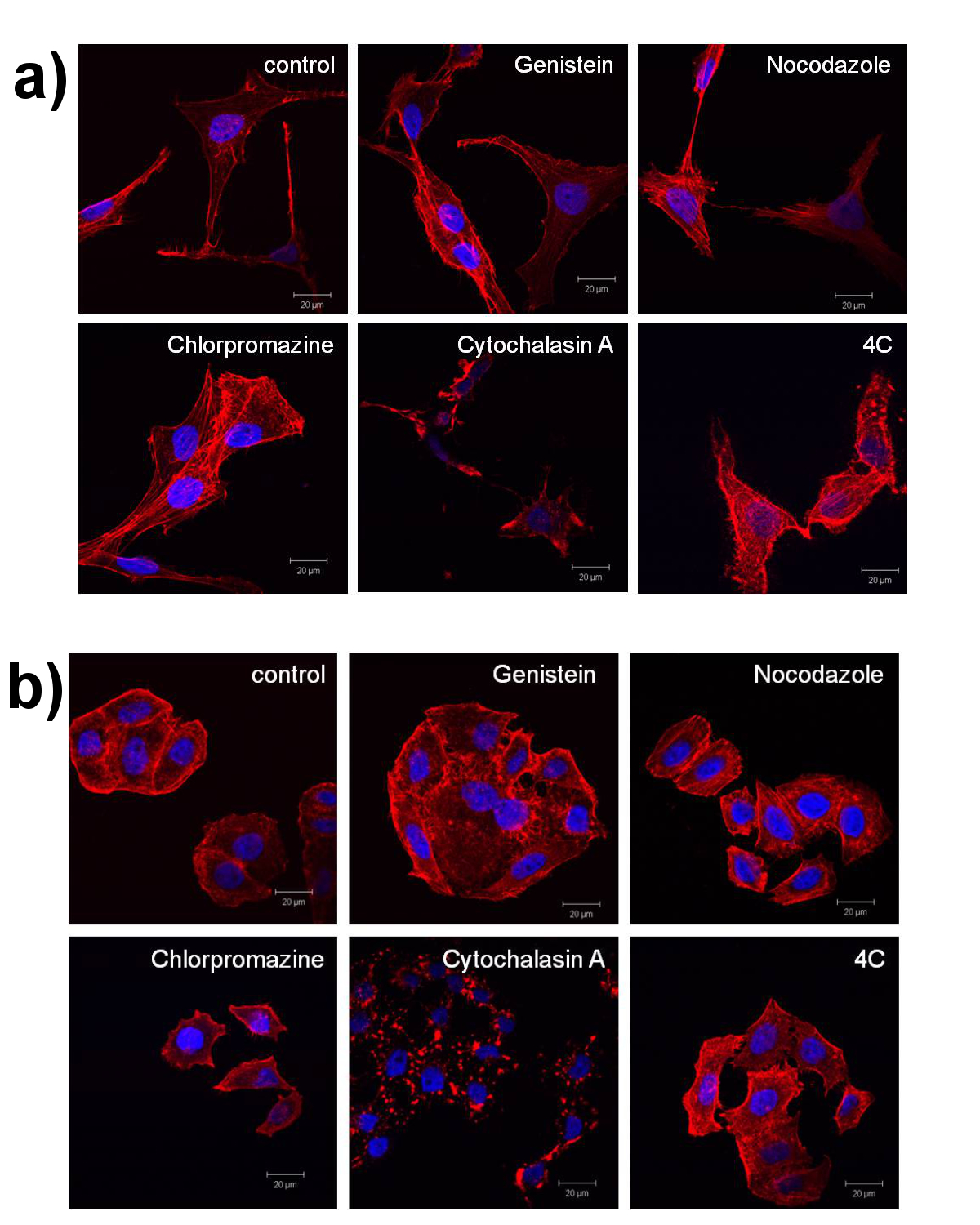

Supplement: Figure S3 — Confocal images of a) 1321N1 and b) HeLa cells, showing their F-actin morphology, after incubation with the different inhibitors at 4°C for 2 h30 min (Blue DAPI stained nuclei, Texas red-phalloidin stained actin filaments. Magnification 63X). The scale bar corresponds to 20 µm. (TIF) [file pone.0024438.s003.tif]

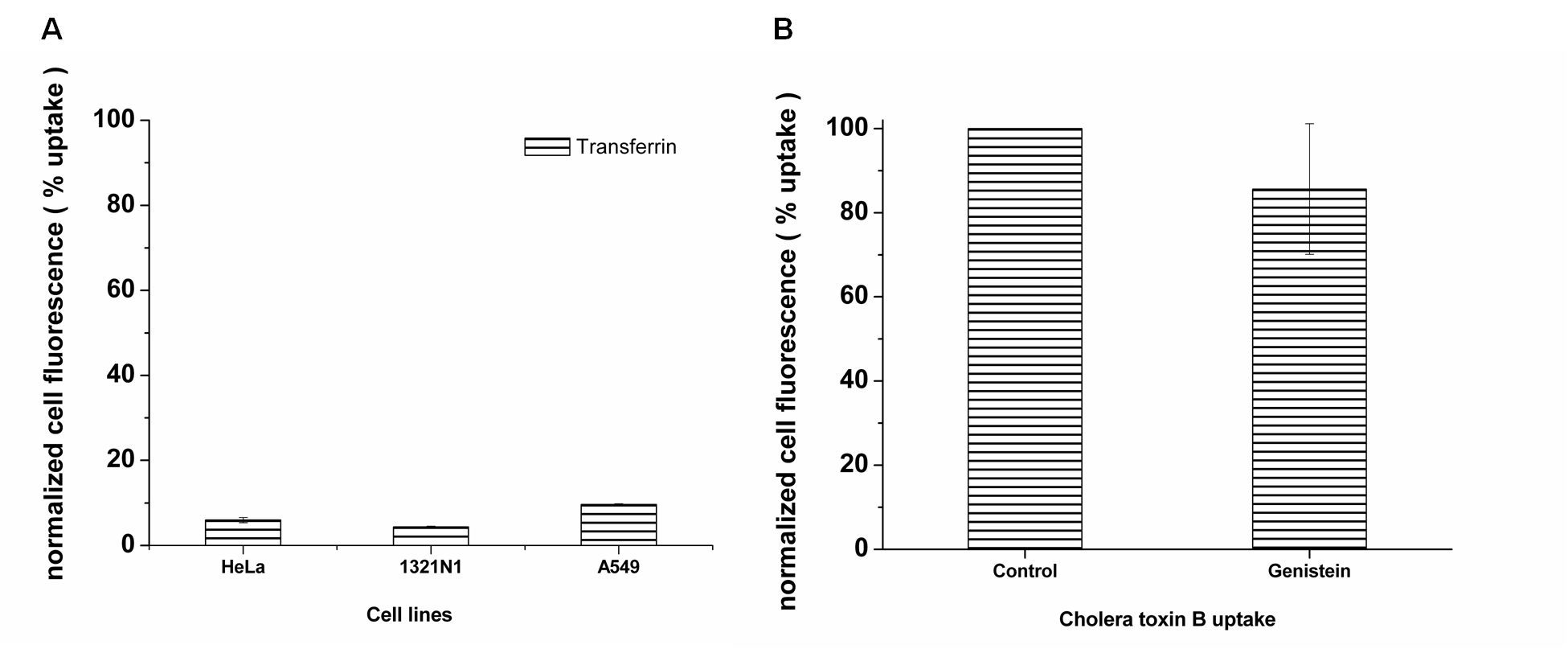

Supplement: Figure S4 — a) Effect of chlorpromazine on internalization of transferrin into different cells. Cells were pre-treated with chlorpromazine for 30 min, followed by 10 min of exposure to Alexa fluor® 488 labelled transferrin in the presence of chlorpromazine, before being fixed and analyzed by FACS. b) Effect of genistein on internalization of cholera toxin B into A549 cells. Cells were pre-treated with genistein for 30 min, followed by 20 min of exposure to Alexa fluor® 488 labelled cholera toxin B in the presence of genistein, before being fixed and analyzed by FACS. Mean values and standard deviations of duplicate samples are given. Results are reported as % uptake relative to the control cells which were not treated with the inhibitor. (TIF) [file pone.0024438.s004.tif]
